# ^68^Ga-FAPI-46 PET/CT in the evaluation of gliomas: comparison with ^18^F-FDG PET/CT and contrast-enhanced MRI

**DOI:** 10.7150/thno.103399

**Published:** 2024-10-21

**Authors:** Dan Ruan, Jianping Sun, Chengkun Han, Jiayu Cai, Lingyu Yu, Liang Zhao, Yizhen Pang, Changjing Zuo, Long Sun, Zhanxiang Wang, Guowei Tan, Xiaobo Qu, Haojun Chen

**Affiliations:** 1 Department of Nuclear Medicine and Minnan PET Center, Xiamen Key Laboratory of Radiopharmaceuticals, the First Affiliated Hospital of Xiamen University, School of Medicine, Xiamen University, Xiamen, China.; 2 National Institute for Data Science in Health and Medicine, Department of Electronic Science, Intelligent Medical Imaging R&D Center, Fujian Provincial Key Laboratory of Plasma and Magnetic Resonance, Xiamen University, Xiamen, China.; 3 Department of Radiology, the First Affiliated Hospital of Xiamen University, School of Medicine, Xiamen University, Xiamen, China.; 4 Department of Nuclear Medicine, Changhai Hospital, Naval Medical University, Shanghai, China.; 5 Department of Neurosurgery, Xiamen Key Laboratory of Brain Center, the First Affiliated Hospital of Xiamen University, School of Medicine, Xiamen University, Xiamen, China.; 6 Xiamen Key Laboratory of Rare Earth Photoelectric Functional Materials, Xiamen Institute of Rare Earth Materials, Haixi Institute, Chinese Academy of Sciences, Xiamen, China.

**Keywords:** ^68^Ga-FAPI-46 PET/CT, ^18^F-FDG, CE-MRI, recurrent detection

## Abstract

***Rationale:*** This study compared ^68^Ga-FAPI-46 PET/CT, ^18^F-fluorodeoxyglucose (FDG) PET/CT, and contrast-enhanced MRI (CE-MRI) for glioma imaging, classification, and recurrence detection and explored PET parameters and molecular pathological profiles.

***Methods:*** Between June 2020 and June 2024, we prospectively enrolled patients with space-occupying lesions in the brain or previously treated gliomas. All patients underwent sequential CE-MRI, ^68^Ga-FAPI-46, and ^18^F-FDG PET/CT. Diagnostic accuracy was assessed based on a reference standard, and PET parameters were analysed for correlations with WHO grading and molecular characteristics.

***Results:*** Forty-eight patients (median age, 51 years; 32 men) with 40 confirmed gliomas were enrolled. For primary tumour diagnosis, the sensitivity of ^68^Ga-FAPI-46 PET/CT was equivalent to CE-MRI (95% vs. 100%, *P* = 0.99) and ^18^F-FDG PET/CT (95% vs. 77%, *P* = 0.13). ^68^Ga-FAPI-46 uptake was higher in grade IV than in grade I-II gliomas (5.03 vs. 1.14, *P* = 0.02). ^68^Ga-FAPI-46 PET/CT showed significantly higher maximum standardized uptake value and tumour-to-background ratio (TBR) in recurrent tumours than in treatment-related changes and demonstrated favourable sensitivity and specificity for detecting recurrent gliomas, though not significantly superior to ^18^F-FDG PET/CT (sensitivity: 100% vs. 85%, *P* = 0.48; specificity: 100% vs. 80%,* P* = 0.99) and CE-MRI (sensitivity: 100% vs. 100%, *P* = NA; specificity: 100% vs. 40%,* P* = 0.25). Glial fibrillary acidic protein-mutant gliomas exhibited higher ^68^Ga-FAPI-46 uptake than wild-type gliomas.

***Conclusion:*** ^68^Ga-FAPI-46 PET/CT outperformed ^18^F-FDG and CE-MRI in diagnosing glioma recurrence, although the results were not statistically significant. For primary glioma diagnosis, ^68^Ga-FAPI-46 PET/CT, despite having a better TBR, did not surpass ^18^F-FDG PET/CT and CE-MRI in terms of sensitivity and specificity. However, ^68^Ga-FAPI-46 PET/CT is superior to ^18^F-FDG for visualizing and classifying gliomas.

## Introduction

Gliomas are the most common primary central nervous system (CNS) malignant tumours, treated with surgical resection, radiation therapy, and chemotherapy 1, 2. Accurate imaging diagnosis and monitoring of tumour recurrence are crucial for treatment implementation and patient prognosis 3.

Contrast-enhanced (CE)-MRI is the standard imaging modality for diagnosing primary and recurrent gliomas. Among the positron emission tomography (PET) tracers, ^18^F-fluorodeoxyglucose (FDG) is the most commonly used; however, its high physiological uptake in normal brain tissues lowers its sensitivity for small brain tumours 4. Amino acid-based PET tracers such as ^11^C-MET and ^18^F-FET exhibit higher uptake in tumours with lower distribution in normal brain tissue, thereby resulting in a higher tumour-to-background ratio (TBR), offering a superior diagnostic advantage over ^18^F-FDG 4. However, ^11^C-MET is limited by its short half-life (20 min). Although ^99m^Tc-MET is an option, its resolution is significantly compromised compared to PET imaging, thus lowering its diagnostic accuracy 5. Recently, ^18^F-fluoroethyl-L-tyrosine (FET) has been fast-tracked for approval by the Food and Drug Administration as a tracer for characterizing glioma progression or recurrence 6. A robust association exists between ^18^F-FET uptake and CE-MRI. Additionally, ^18^F-FET aids in identifying true progression or treatment-induced changes in gliomas that cannot be recognized by CE-MRI 7. However, the long retention time of ^18^F-FET in the blood may lead to increased physiological uptake of ^18^F-FET in vascular malformations and dural venous sinuses, complicating the differentiation between metabolically active tumours and blood vessels 8. Additionally, approximately 30% of low-grade gliomas and 10% of high-grade gliomas are ^18^F-FET negative, with unclear underlying mechanisms 9, 10, prompting exploration of alternative imaging technologies.

Fibroblast-activation protein (FAP) is expressed in gliomas, with significantly higher expression in high-grade gliomas than in low-grade ones 11. FAP expression is undetectable in glioblastoma cells *in vitro* but is expressed *in vivo*, including in tumour cells 12. Single-nucleus RNA sequencing revealed that FAP is expressed in tumour cells and surrounding tumour vascular cells in human glioblastomas 13.

Studies on fibroblast-activation protein inhibitor (FAPI)-based PET tracers in gliomas are limited. Röhrich *et al.* demonstrated a significant increase in ^68^Ga-FAPI-04 uptake in isocitrate dehydrogenase (IDH) wild-type glioblastomas and high-grade IDH-mutant astrocytoma but not in diffuse astrocytoma 12. Yao's study assessed the diagnostic value of ^18^F-FAPI-04 in a small number of patients with glioblastomas and revealed excellent TBR and clear lesion visualisation 14. Therefore, we hypothesized that ^68^Ga-FAPI-46 PET/CT may demonstrate high diagnostic accuracy for primary and recurrent gliomas. This study aimed to assess the clinical feasibility of ^68^Ga-FAPI-46, a pan-cancer PET imaging agent, for glioma diagnosis, classification, and recurrence detection. We compared the results with standard-of-care examinations, including ^18^F-FDG PET/CT and CE-MRI. Additionally, we explored the correlation between PET parameters and the molecular pathological profiles of gliomas.

## Methods

### Patients

This prospective study was approved by the Institutional Review Board of our hospital (IRB XMYY-2020KY063) and was registered at ClinicalTrials.gov (NCT04416165). Forty-nine patients with suspected primary and recurrent gliomas were enrolled between June 2020 and June 2024. Written informed consent was obtained from all participants. All patients underwent CE-MRI, ^18^F-FDG, and ^68^Ga-FAPI-46 PET/CT within 2 weeks. The inclusion criteria were patients who: (1) underwent PET/CT to assess the nature of a space-occupying brain lesion; (2) previously treated glioblastoma and underwent PET/CT to evaluate suspected recurrence; (3) no prior anti-tumour treatment within 4 weeks of PET imaging; and (4) able to complete CE-MRI, ^18^F-FDG, and ^68^Ga-FAPI-46 PET/CT in 2 weeks. The exclusion criteria were patients with (1) known metastases from other tumours, (2) other primary CNS disease and infectious diseases, and (3) pregnancy.

### Synthesis of ^18^F-FDG and ^68^Ga-FAPI-46

^18^F-FDG was manufactured in accordance with the standard method described by our laboratory using the coincidence ^18^F-FDG synthesis module (TracerLab Fx FDG; GE Healthcare, Milwaukee, Wis, USA). The FAPI-46 precursor was obtained from CSBio (Shanghai, China) for research and development purposes. ^68^Ga-FAPI-46 was prepared according to a previous protocol 15. Radiochemical purity exceeded 95% for both ^18^F-FDG and ^68^Ga-FAPI-46, and the final product was diluted with saline and sterilized using a 0.22-μm Millipore filter (EMD Millipore, Billerica, Mass) into a sterile multidose syringe. The sterility tests were performed in-house in the radiochemistry facility.

### PET/CT and MRI image acquisition

^68^Ga-FAPI-46 PET/CT scans were performed within 2 weeks of ^18^F-FDG PET/CT. The median interval between the two examinations was 2 days (1-6 days). Intravenous injection doses of ^18^F-FDG and ^68^Ga-FAPI-46 were calculated based on participant weight (^18^F-FDG: 3.7 MBq [0.1 mCi]/kg; ^68^Ga-FAPI-46: 1.8-2.2 MBq [0.05-0.06 mCi]/kg). One hour after the intravenous injection, the participants underwent PET/CT examinations using a hybrid PET/CT scanner (Discovery MI; GE Healthcare). All acquired data were transferred to the Advantage Workstation version AW 4.7 (GE Healthcare) and reconstructed using the Bayesian-penalised likelihood reconstruction algorithm (Q.clear; GE Healthcare).

All patients underwent CE-MRI as standard-of-care imaging. Imaging sequences included T1-weighted (T1W), T2-weighted, T2-weighted fluid-attenuated inversion recovery (FLAIR), contrast-enhanced T1W, postcontrast T1-weighted volume imaging (MPRAGE), and contrast-enhanced FLAIR. Contrast-enhanced images were obtained following the intravenous injection of gadolinium sulphate according to standard procedures.

### PET/CT and MRI image analysis

MRI images were used as anatomical references for the qualitative analysis of PET images. Tumour activity was visually assessed by two experienced nuclear medicine physicians (C.H. and W.H.), who compared the areas of highest ^68^Ga-FAPI-46/^18^F-FDG uptake within the tumour to the uptake in normal grey and white matter on the contralateral side. For lesions without normal contralateral tissue (such as brainstem gliomas), normal grey and white matter at the level of the centrum semiovale was used for comparison. For ^68^Ga-FAPI-46 PET, a positive result was defined as tracer uptake in suspicious lesions higher than the background uptake in normal brain parenchyma. ^18^F-FDG uptake was scored as follows: 0 = uptake less than that in white matter (WM); 1 = uptake equal to that in WM; 2 = uptake between WM and the cerebral cortex; 3 = uptake equal to or greater than that in the cerebral cortex. Gliomas with a visual score of 0 or 1 were classified as ^18^F-FDG negative, while gliomas with a visual score of 2 or 3 were classified as ^18^F-FDG positive. Two experienced radiologists (H.C. and C.J.) interpreted the brain MRI images. On MRI, lesions showing enhancement on contrast-enhanced sequences, hyperintensity on FLAIR sequences, or abnormal ratios on spectroscopy were considered positive for malignancy.

For quantitative analysis, the PET and MRI data were registered using the Advanced Normalization Tools (ANTs for Linux, version 2.5.3). Regions of interest (ROIs) were placed in areas of abnormal signal on the MR images and then transferred to the registered ^68^Ga-FAPI-46 and ^18^F-FDG PET scans. All ROIs were carefully positioned to avoid areas of necrosis, haemorrhage, or normal tissue. A 10×10 mm circular ROI was manually placed in the unaffected contralateral frontal cortex for the reference ROI. The TBR was calculated by dividing the maximum standardized uptake value (SUVmax) within the tumour ROI by the mean SUV in the ROI of the contralateral normal brain parenchyma.

### Reference standard

Tissue pathology results obtained from surgery or biopsy serve as the gold standard for the final diagnosis. In some patients with suspicious recurrence whose tissue biopsy may not be applicable, clinical follow-up, including repeated MRI and clinical outcomes (for at least 6 months), was used to validate the PET/CT findings. Disease-related adverse events (death or neurological deterioration) and progressive disease on follow-up MRI were considered positive for recurrence.

Histopathological diagnosis of gliomas was performed according to the WHO 2016 classification of CNS tumours. Immunohistochemistry (IHC) and direct gene sequencing were performed on formalin-fixed paraffin-embedded tumour tissues obtained surgically. The IDH mutation status was determined by detecting the IDH1-R132H point mutation using monoclonal antibodies in the IHC of glioma specimens. Patients who were positive for IDH1 were classified into the IDH mutation group. Genomic sequence analysis was performed on negative samples to identify atypical IDH1 or IDH2 genes. In addition to the IDH status, the molecular expression of glial fibrillary acidic protein (GFAP), epidermal growth factor receptor (EGFR), alpha-thalassemia mental retardation X-linked (ATRX), nuclear factor-κB (NF), Ki-67, and TP53 was analysed by IHC. Detailed methods for these molecular analyses have been described previously 16.

### Statistical analysis

Statistical analyses and data visualisation were performed using R software (Windows version 4.2.2). Descriptive statistics summarised the patients' basic characteristics, with continuous variables presented as medians and interquartile ranges (IQR) and categorical variables as frequencies or percentages. *T-tests* were used for normally distributed parameters; otherwise, the Wilcoxon rank-sum test was used. The diagnostic performance differences between imaging methods were assessed using the McNemar test. Pearson's correlation coefficient was used for correlation analysis between variables. Statistical significance was set at *P* < 0.05.

## Results

### Patients' characteristics

**Figure 1:**
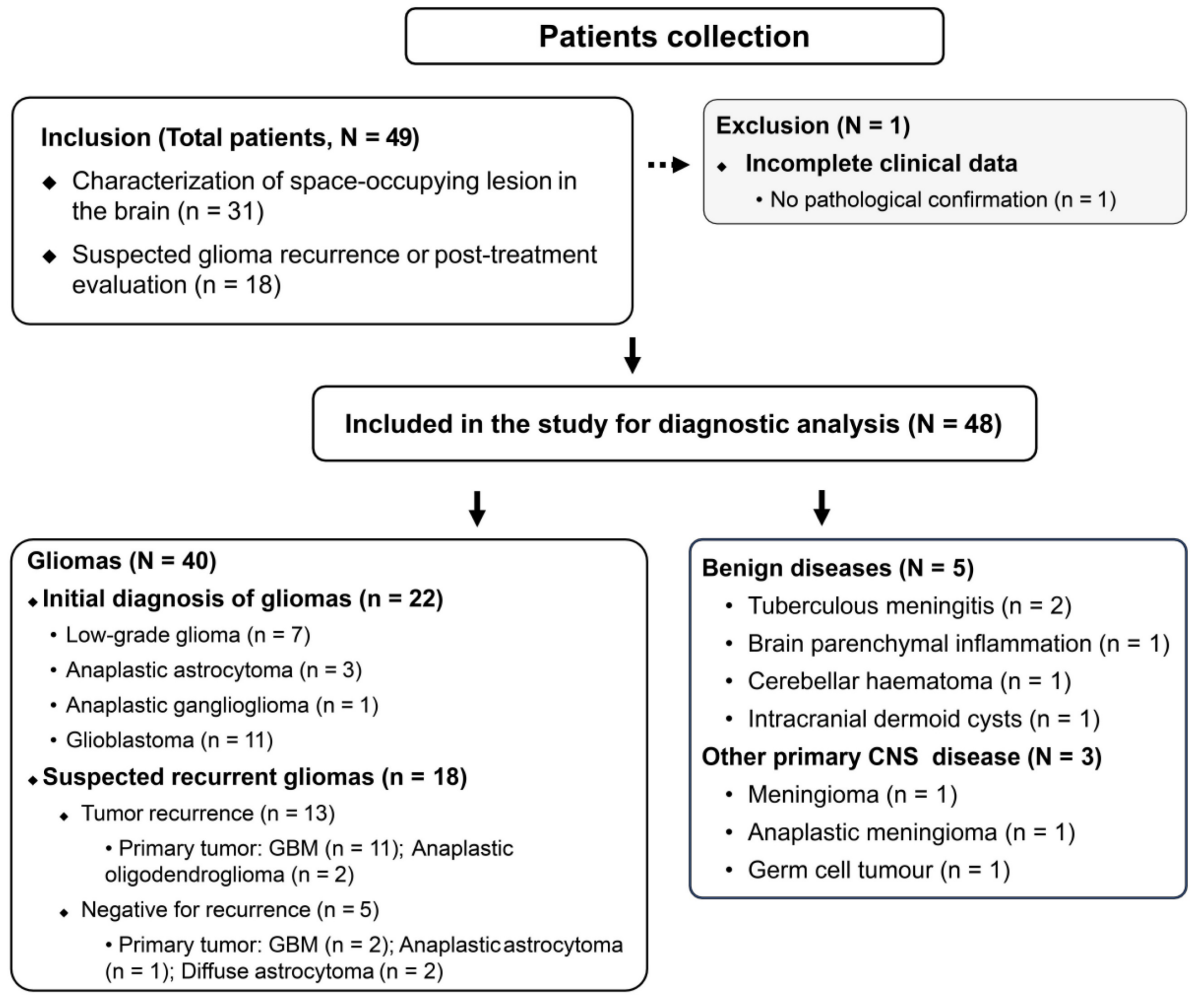
Flow diagram of study inclusion and exclusion criteria.

During a median follow-up of 25.1 months (IQR, 14.8-39.4 months), 48 participants were prospectively enrolled in the study. One patient was excluded owing to a lack of pathological confirmation and a loss to follow-up. A schematic representation of the study design is shown in Figure 1. The median patient age was 51 (IQR, 44-67) years and included 32 men. The indications for PET/CT were the characterization of space-occupying brain lesions (63%, 30/48 patients) and the evaluation of suspected recurrence (37%, 18/48 patients; Table 1).

Among the 30 treatment-naive patients with space-occupying brain lesions, five patients were diagnosed with non-glioma lesions, which included tuberculous meningitis (n = 2), brain parenchymal inflammation (n = 1), cerebellar haematoma (n = 1), and intracranial dermoid cysts (n = 1; Figure S1). Three patients were diagnosed with other primary CNS diseases (germ cell tumour, meningioma, and anaplastic meningioma, respectively). Seven patients were diagnosed with low-grade gliomas (WHO grade I-II), 4 with WHO grade III gliomas (anaplastic astrocytoma and anaplastic ganglioglioma), and 11 with WHO grade IV gliomas (glioblastomas).

Among the 18 patients with suspected recurrent tumours, 13 were positive for viable tumours (five patients were histologically confirmed), while the remaining five were negative for recurrence. All recurrent tumours were initially diagnosed as primary high-grade tumours (11 glioblastomas and two anaplastic oligodendrogliomas). Two of the five negative patients had primary low-grade gliomas (diffuse astrocytoma), and three had primary high-grade gliomas (two glioblastomas and one anaplastic astrocytoma).

### Comparison of PET and CE-MRI for the diagnosis of primary gliomas

Histopathological results of the space-occupying brain lesions were available for all patients. Three patients with other primary CNS diseases were excluded from diagnostic analysis. The final cohort for initial diagnosis included 22 patients with treatment-naïve gliomas and 5 patients with non-glioma lesions. ^68^Ga-FAPI-46 PET/CT showed a sensitivity of 95% (21/22) for detecting primary gliomas, whereas ^18^F-FDG PET/CT and CE-MRI showed sensitivities of 77% (17/22) and 100% (22/22), respectively. There were no significant differences regarding glioma detectability between ^68^Ga-FAPI-46 and ^18^F-FDG PET/CT (*P* = 0.13), and no differences between ^68^Ga-FAPI-46 and CE-MRI (*P* = 0.99; Table 2). The specificity and accuracy of ^68^Ga-FAPI-46 PET/CT were 40% (2/5) and 85% (23/27), respectively. For ^18^F-FDG PET/CT, the specificity and accuracy were 100% (5/5) and 81% (22/27), respectively, and 20% (1/5) and 85% (23/27) for CE-MRI.

**Figure 2:**
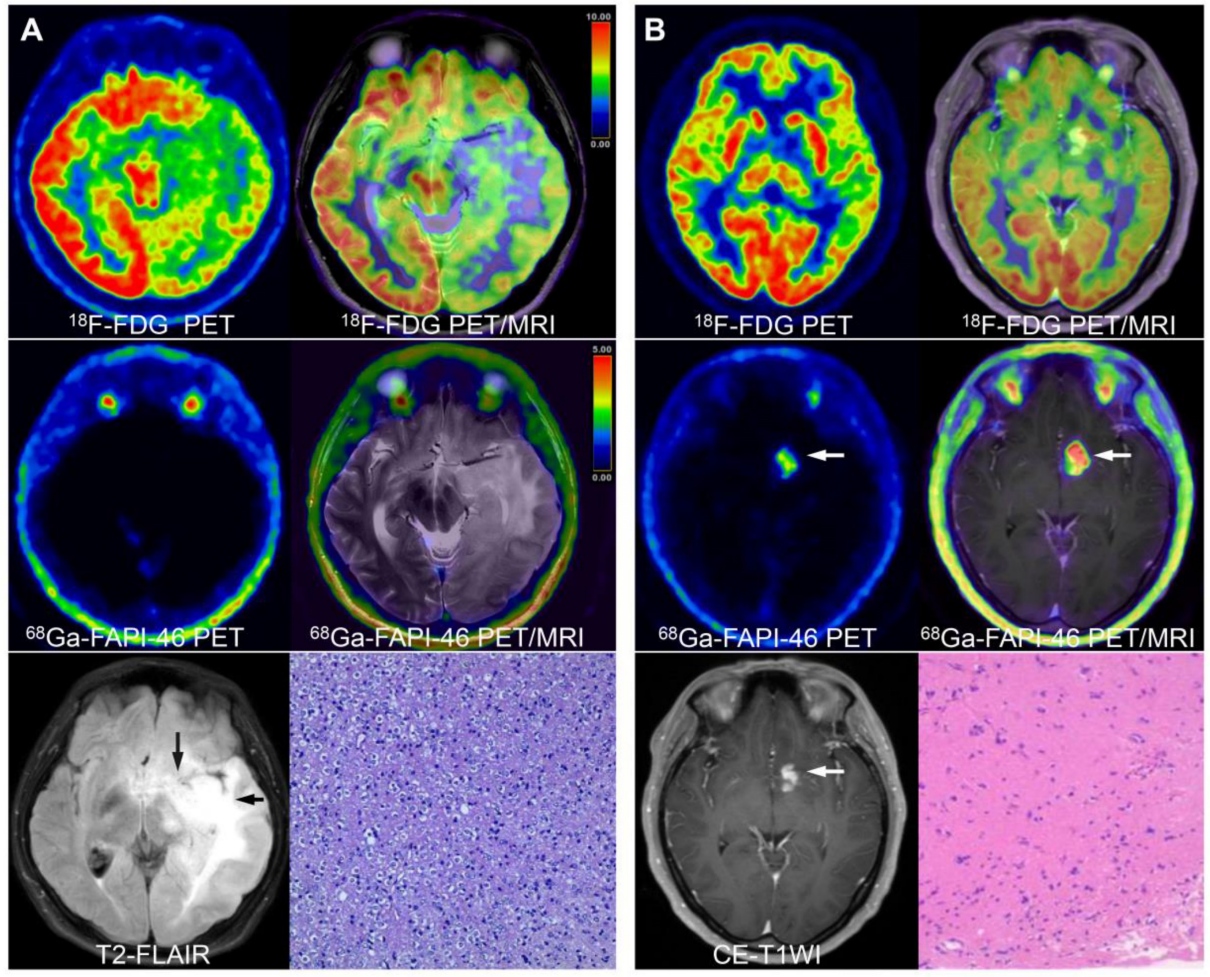
True-positive and false-negative findings of low-grade gliomas on ^68^Ga-FAPI-46 PET (lesions are indicated by arrows). A 17-year-old male presented with recurrent vomiting. No significant tracer uptake was observed on ^68^Ga-FAPI-46 PET. ^18^F-FDG PET showed diffusely reduced glucose metabolism in the left cerebral parenchyma. T2-FLAIR images revealed abnormal high signals in the left temporal lobe, basal ganglia, insula, thalamus, and brainstem. The final pathological diagnosis was diffuse astrocytoma **(A)**. A 38-year-old female presented with a headache. No significant abnormalities were observed on ^18^F-FDG PET. However, on ^68^Ga-FAPI-46 PET and PET/MRI fusion images, intense radiotracer uptake was observed in the left basal ganglia, which showed abnormal enhancement on CE-MRI. The final pathological diagnosis was a low-grade ganglioglioma **(B)**.

^68^Ga-FAPI-46 PET/CT showed one false-negative result (diffuse astrocytoma [Figure 2A]) and three false-positive findings (two tuberculous meningitis and one brain parenchymal inflammation Figure S1). In contrast, ^18^F-FDG PET/CT showed five false-negative findings (including four low-grade gliomas and one glioblastoma). Four false-positive (one cerebellar haematoma, two tuberculous meningitis, and one brain parenchymal inflammation) findings were noted regarding CE-MRI (Table S1).

### Comparison of ^18^F-FDG and ^68^Ga-FAPI-46 uptake in primary gliomas

**Figure 3:**
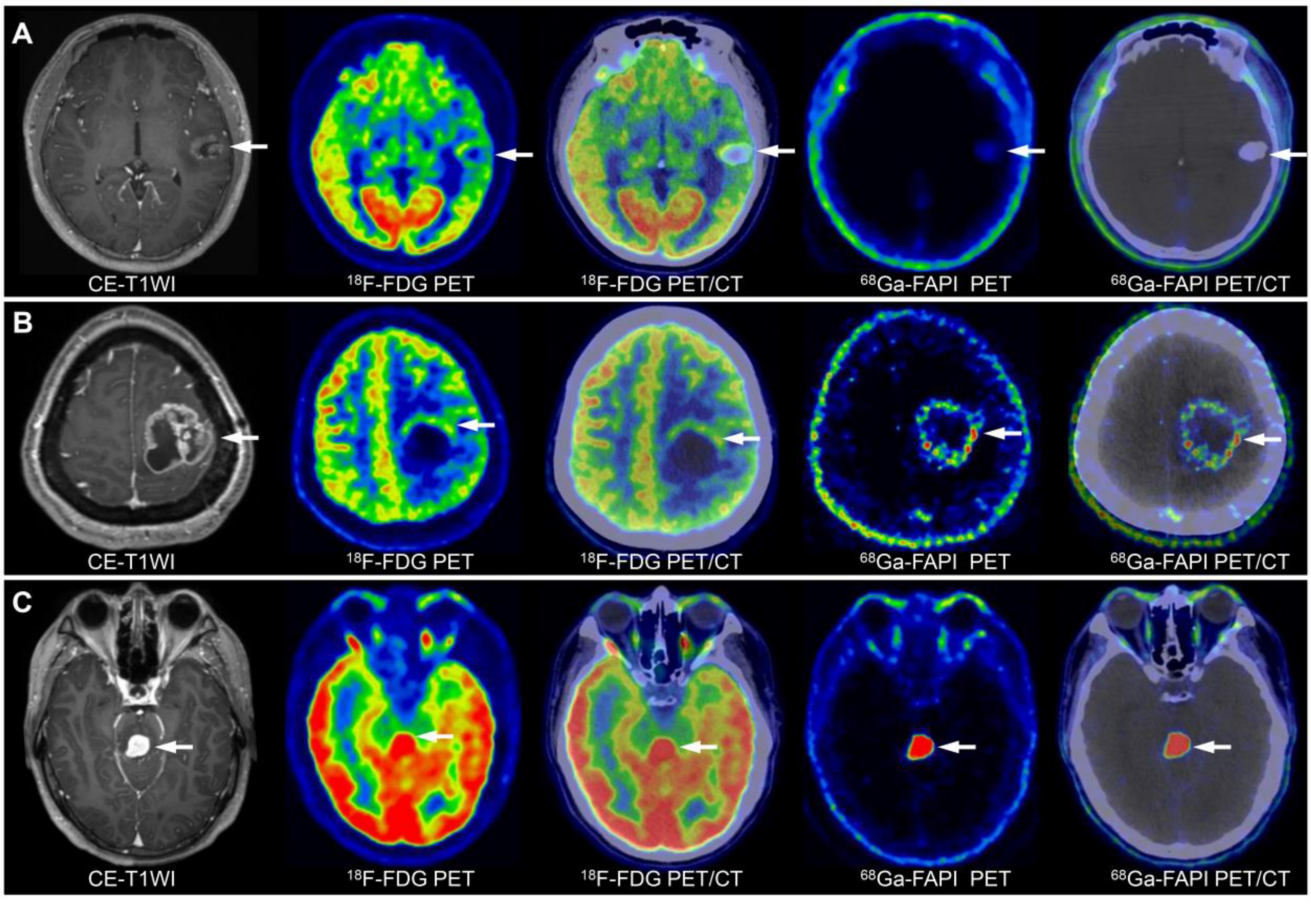
Image characterisation of different WHO grades of gliomas on ^68^Ga-FAPI-46 PET, ^18^F-FDG PET, and CE-MRI (tumour lesions are indicated by the arrows). A 15-year-old male with a low-grade oligodendroglioma in the left temporal lobe showed mild ^18^F-FDG and ^68^Ga-FAPI-46 uptake **(A)**. A 45-year-old female with a WHO grade III glioma in the left parietal lobe exhibited ring-shaped elevated ^68^Ga-FAPI-46 uptake, with locally uneven increased ^18^F-FDG at the margin **(B)**. A 36-year-old male with a WHO grade IV glioma in the brainstem demonstrated significantly increased tracer uptake on both ^68^Ga-FAPI-46 and ^18^F-FDG PET/CT **(C)**.

Grade I-II gliomas showed significantly higher uptake of ^18^F-FDG than ^68^Ga-FAPI-46 (7.74 vs. 1.14, *P* < 0.001); however, the TBR from ^18^F-FDG PET/CT was significantly lower than that from ^68^Ga-FAPI-46 PET/CT (1.41 vs. 4.57, *P* = 0.002). Therefore, ^68^Ga-FAPI-46 demonstrated advantages over ^18^F-FDG for lesion visualisation (Figure 2B). Grade III gliomas exhibited high ^18^F-FDG uptake (median SUVmax = 11.60) but relatively low image contrast with a TBR of 1.45 (IQR: 1.37-1.86). Glioblastomas (grade IV) showed intense uptake of both ^18^F-FDG and ^68^Ga-FAPI-46. The SUVmax derived from ^18^F-FDG PET was significantly higher than that from ^68^Ga-FAPI-46 PET (10.33 vs. 5.03, *P* = 0.001). However, ^68^Ga-FAPI-46 PET, with a high TBR (median: 19.24, IQR: 13.02-46.04), visualised the tumour clearly, whereas ^18^F-FDG PET showed a significantly lower TBR (median 1.73, IQR 1.36-2.38). The PET semiquantitative results are summarised in Table 3, and the representative images are shown in Figures 3 and S2.

On ^18^F-FDG PET/CT, although grade III (median: 11.60) and IV (median: 10.33) gliomas had higher SUVmax than grade I-II gliomas (median: 7.74; III vs. I-II, *P =* 0.16; IV vs. I-II, *P =* 0.18), grade IV gliomas (median: 1.73) showed slightly higher TBR than grade III (median: 1.45) and grade I-II gliomas (median 1.41; IV vs. III, *P =* 0.85; IV vs. I-II, *P =* 0.15), and the differences were not significant. On ^68^Ga-FAPI-46 PET, grade IV gliomas exhibited a significantly higher SUVmax than grade I-II (5.03 vs. 1.14, *P =* 0.02). Therefore, ^68^Ga-FAPI-46 PET may be more suitable than ^18^F-FDG PET for grading gliomas. Grade III gliomas showed higher ^68^Ga-FAPI-46 uptake (4.76 vs. 1.14, *P =* 0.07) and TBR (26.28 vs. 4.57, *P =* 0.11) than grade I-II gliomas, although the differences were not statistically significant. Overall, across all WHO grades, ^18^F-FDG PET demonstrated a higher SUVmax (10.11 vs. 4.41, *P* < 0.001) but a lower TBR (1.52 vs. 17.50, *P* < 0.001) than ^68^Ga-FAPI-46 (Table 3).

### Comparative diagnostic accuracy of recurrent gliomas

**Figure 4:**
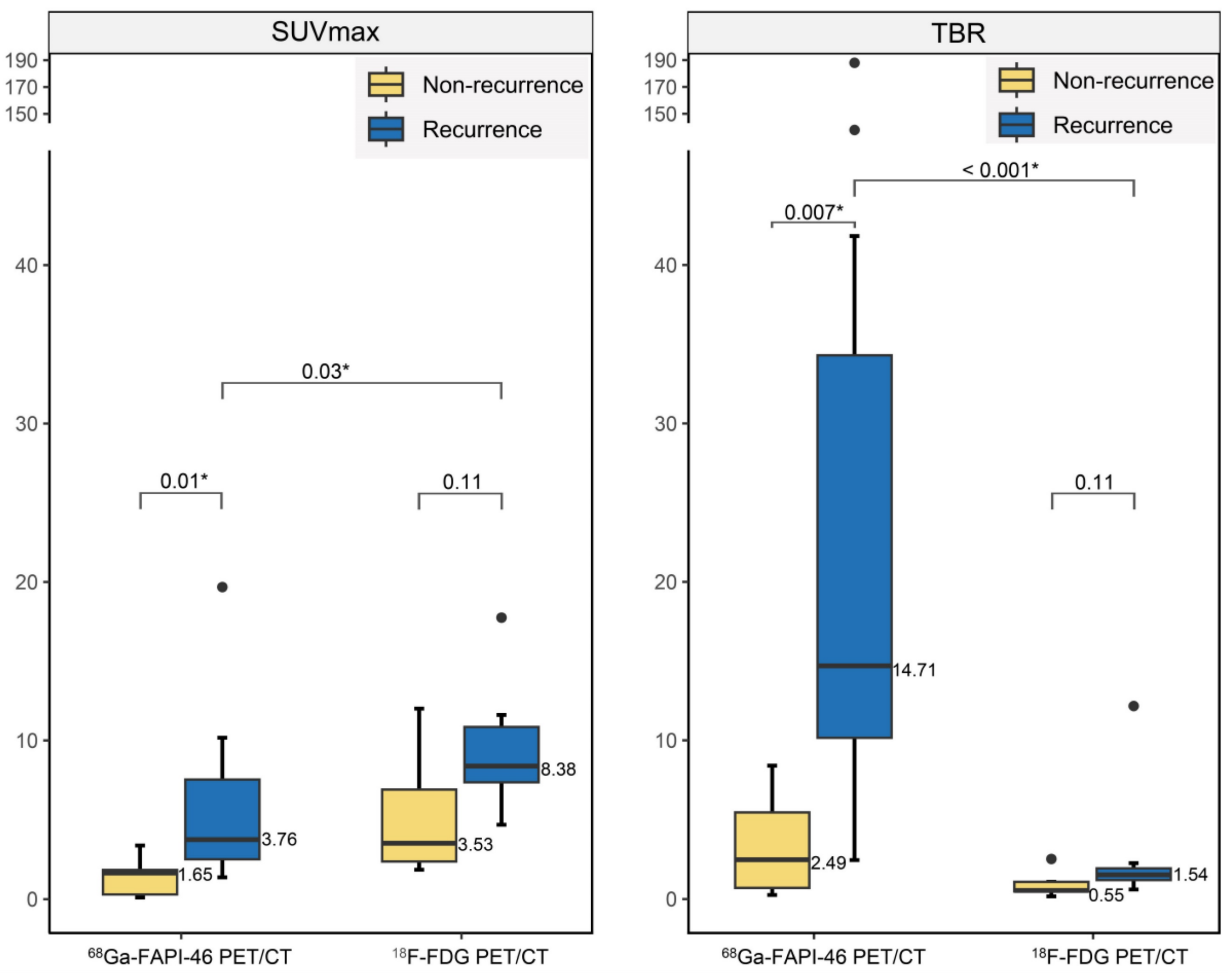
Differences in radiotracer uptake between recurrent and non-recurrent gliomas.

**Figure 5:**
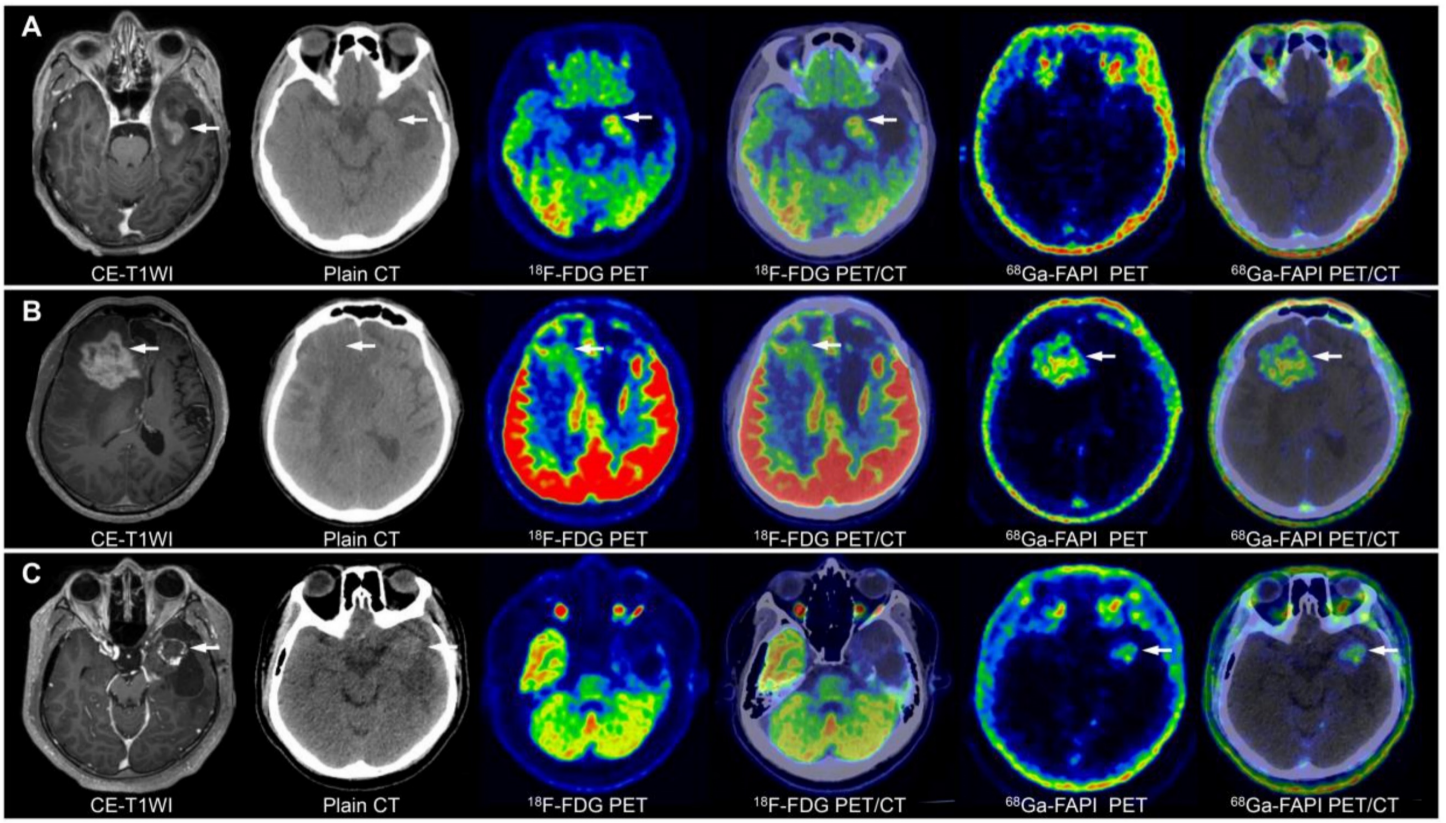
PET/CT and CE-MRI for the detection of glioma recurrence (lesions are indicated by arrows). A 44-year-old male with a history of surgery and radiotherapy for anaplastic astrocytoma had a suspicious recurrent lesion in the left temporal lobe on CE-MRI, with a significantly increased ^18^F-FDG uptake but negative ^68^Ga-FAPI-46 uptake. The follow-up MRI revealed loss of enhancement of this lesion, which confirmed no evidence of tumour recurrence **(A)**. Another 44-year-old male with recurrent anaplastic oligodendroglioma in the right frontal lobe after surgery showed mildly increased ^18^F-FDG uptake and significantly increased ^68^Ga-FAPI-46 uptake, with the lesion visible on ^68^Ga-FAPI-46 PET/CT **(B)**. A 30-year-old male with recurrent glioblastoma in the temporal lobe after surgery exhibited contrast-enhanced foci on CE-T1WI in the surgical area, with mild tracer uptake on ^18^F-FDG PET/CT and significantly increased tracer uptake on ^68^Ga-FAPI-46 PET/CT **(C)**.

^18^F-FDG PET showed no significant differences in SUVmax (8.38 vs. 3.53, *P =* 0.11) or TBR (1.54 vs. 0.55, *P =* 0.11) between recurrent tumours and post-treatment changes (treatment-related changes without residual or recurrent glioma; Figure 4). However, ^68^Ga-FAPI-46 PET demonstrated significantly higher SUVmax (3.76 vs. 1.65, *P =* 0.01) and TBR (14.71 vs. 2.49, *P =* 0.007) in recurrent tumours compared to postoperative changes (pseudoprogression and radiation necrosis). Although the uptake of both tracers increased in recurrent tumours, ^18^F-FDG exhibited a higher SUVmax than ^68^Ga-FAPI-46 (SUVmax, 8.38 vs. 3.76, *P =* 0.03). ^68^Ga-FAPI-46 PET showed a significantly higher TBR for recurrence than ^18^F-FDG (TBR, 14.71 vs. 1.54, *P* < 0.001), allowing a more precise visualisation of recurrent lesions (Figure 5).

The sensitivity of ^68^Ga-FAPI-46 PET/CT (100% [13/13]) was comparable to that of CE-MRI (100% [13/13]) and slightly higher than that of ^18^F-FDG PET/CT (85% [11/13]), though these differences were not statistically significant (*P* = 0.48; Table 4). The specificity of ^68^Ga-FAPI-46 PET/CT (100% [5/5]) surpassed that of ^18^F-FDG PET/CT (80% [4/5]) and CE-MRI (40% [2/5]), although the differences were not statistically significant (*P* = 0.99 for ^68^Ga-FAPI-46 vs. ^18^F-FDG; *P* = 0.25 for ^68^Ga-FAPI-46 PET/CT vs. CE-MRI). Similarly, the accuracy of ^68^Ga-FAPI-46 PET/CT (100% [18/18]) was higher than that of ^18^F-FDG PET/CT (83% [15/18]) and CE-MRI (83% [15/18]), although the difference was not statistically significant (*P* = 0.99 for ^68^Ga-FAPI-46 vs. ^18^F-FDG; *P* = 0.25 for ^68^Ga-FAPI-46 PET/CT vs. CE-MRI). Specifically, ^18^F-FDG PET/CT produced one false-positive result, with follow-up MRI showing lesion disappearance and two false negatives owing to low TBR (Figure 5). CE-MRI resulted in three false-positive results: two cases of abnormal enhancement with no changes on follow-up MRI and one case where the lesion disappeared.

### Association between molecular features and PET parameters

Fifteen patient samples were tested for p53 and Ki-67 indices, 13 for IDH, 13 for GFAP, 10 for ATRX, 6 for NF, and 5 for EGFR. No significant correlations were observed between the Ki-67 index, TP53 expression, and ^68^Ga-FAPI/^18^F-FDG PET parameters (Table S2; Figure S3). GFAP mutant gliomas exhibited significantly higher ^68^Ga-FAPI-46 uptake than wild-type (4.86 vs. 1.35, *P* = 0.01). Tracer uptake and TBR did not differ significantly between gliomas with or without molecular changes in ATRX, NF, or EGFR (*P* > 0.05). The data for IDH were insufficient to draw conclusive results (Figure S4).

## Discussion

^68^Ga-FAPI-46 is a promising novel PET tracer for imaging various tumours; however, studies on gliomas are relatively rare. This study assessed the clinical feasibility of ^68^Ga-FAPI-46 PET/CT for evaluating gliomas. ^68^Ga-FAPI-46 PET/CT and CE-MRI demonstrated similar high sensitivity for the initial diagnosis of gliomas. Compared to ^18^F-FDG, ^68^Ga-FAPI-46 PET/CT offers better tumour-to-background contrast, providing glioma delineation and tumour grading advantages. Significant differences in SUVmax and TBR were observed between recurrent and nonrecurrent lesions on ^68^Ga-FAPI-46 PET/CT.

For grade I-II gliomas, ^68^Ga-FAPI-46 PET/CT accurately located and visualised lesions in the majority of patients (6/7, 86%), confirming the findings reported by Röhrich *et al.* 12. However, one patient with diffuse astrocytoma showed false-negative uptake of ^68^Ga-FAPI-46, which is consistent with earlier studies indicating that diffuse astrocytoma showed very few FAP-positive tumour cells and FAP-positive tumour-associated pericytes 17. FAP expression significantly increases with tumour grade 17, 18. In our study, high-grade gliomas exhibited significantly higher ^68^Ga-FAPI uptake than low-grade gliomas, underscoring the potential of ^68^Ga-FAPI-46 PET/CT for tumour grading and providing a non-invasive and intuitive means of predicting patient prognosis 13, 18, 19. High FAP expression promotes invasion, migration, and epithelial-mesenchymal transition in glioblastomas 18. Consequently, assessing ^68^Ga-FAPI-46 uptake levels may discern malignant transformation and enhanced invasiveness in gliomas.

Moreover, FAP has been identified as a target for immunotherapy aimed at selectively destroying glioma cells and their supporting vascular networks 19, as demonstrated in preclinical studies 13, 18. Hence, ^68^Ga-FAPI-46 PET/CT may be valuable for patient screening and evaluating the efficacy of FAP-targeted immunotherapy. Notably, significant tumour growth inhibition was observed with ^177^Lu-EB-FAPI (an FAP-targeted therapeutic radiopharmaceutical) in human glioblastoma xenografts, suggesting the feasibility of FAP-targeted radioligand therapy in refractory and recurrent gliomas in future clinics 20.

Notably, ^68^Ga-FAPI-46 is not a specific imaging agent for imaging gliomas. Nodular or clustered ^68^Ga-FAPI-46 uptake was observed in conditions including inflammatory lesions, tuberculosis, and haemorrhage, and such false-positive findings were also observed in ^18^F-FET PET 7. In one case of cerebral parenchymal inflammation, the lesion range and location depicted on CE-MRI did not correspond with the areas of ^68^Ga-FAPI-46 uptake. This resembled a case of leukoencephalopathy in which increased ^68^Ga-FAPI uptake was located at the periphery of MRI-demonstrated lesions 21. This discrepancy may be because of the difference between the locations of ^68^Ga-FAPI uptake and contrast enhancement in such diseases, which warrants further investigation. Additionally, ^68^Ga-FAPI uptake in tuberculous meningitis has been reported 22, possibly related to repeated brain tissue injury and fibrosis induced by *Mycobacterium tuberculosis* 23. Therefore, false-positive findings caused by increased ^68^Ga-FAPI uptake in some non-malignant diseases should be noted for characterizing space-occupying brain lesions.

Post-therapy changes in treated patients with glioma cannot be reliably differentiated from tumour recurrence. Notably, ^68^Ga-FAPI-46 PET/CT accurately identified post-treatment changes and tumour recurrence. Pseudo-progression and radiation necrosis are characterised by tissue inflammation, oedema, increased vascular permeability, signs of blood-brain barrier (BBB) disruption, and mass effects, which manifest as new or increased contrast enhancement on CE-MRI 24. Compared to amino acid-based radiotracers, CE-MRI showed lower sensitivity and specificity for evaluating recurrent gliomas 5. Combining MR spectroscopy (MRS) to distinguish between treatment-related changes and recurrence is complex and challenging. Recurrence, pseudo-progression, and radiation necrosis exhibit neuronal loss (low NAA), functional abnormalities (high Cho), abnormal cell membranes, and metabolic abnormalities (elevated lactate/lipids). In particular, the ability of MRS to distinguish between necrotic tissue and mixtures of necrotic and tumour tissue is very limited 25. In our study, MRS analysis in two CE-MRI positive cases (Cho/NAA ratios both less than 0.7) suggested radiation-induced brain injury (Figure S5). However, follow-up results showed recurrence with rapid disease progression.

The limitation of ^18^F-FDG PET/CT lies in its difficulty in detecting hypometabolic lesions or lesions of small size (< 20 mm) within strong physiological brain uptake 26. Aside from tumour stroma and tumour cells, fibroblasts in actively remodelling tissues, such as chronic inflammation and fibrotic tissue, also exhibit upregulated FAP expression. This may affect the diagnostic reliability of ^68^Ga-FAPI PET 27. However, unlike other solid tumours, false-positive findings from benign tissue repair or inflammation were not observed in recurrent gliomas. This may be related to the surgery or radiotherapy intervention time, in which no ^68^Ga-FAPI-46 uptake was observed in the early phase of pseudo-progression and radiation necrosis 28. However, larger research cohorts are needed for validation. ^68^Ga-FAPI-46, with its superior imaging characteristics in primary and recurrent gliomas, could complement ^18^F-FDG and amino acid radiotracers (^18^F-FET and ^11^C-MET), especially in patients with suspected tumour recurrence where the specificity of ^18^F-FDG is reduced (owing to inflammation or treatment-related changes).

As depicted in this study and previous investigations 29, ^68^Ga-FAPI-46 may not be able to cross the BBB. For most primary and recurrent gliomas in this study, increased ^68^Ga-FAPI-46 uptake corresponds to CE-MRI findings, indicating that tumour uptake of ^68^Ga-FAPI-46 may also be related to BBB leakage. However, three patients with pseudo-progression showed negative ^68^Ga-FAPI-46 uptake but demonstrated contrast enhancement on the MRI, indicating that ^68^Ga-FAPI-46 uptake is mainly driven by FAP expression in case of BBB leakage. Thus, it is foreseeable that the performance of ^68^Ga-FAPI-46 PET would have been worse if more low-grade, usually CE-negative gliomas had been included. This also implies that the detected differences between tumour grades are likely driven by BBB leakage. Thus, in future studies, increasing the sample size to get a larger, more balanced, and more representative population is necessary. Most importantly, BBB leakage/CE+ should be considered as a covariate. In practical scenarios, using ^68^Ga-FAPI-46 PET/CT to diagnose low-grade gliomas may be most useful in cases where the BBB is disrupted, or standard examinations are inconclusive. Future studies should explore molecular biomarkers and imaging probes that are more sensitive and specific for CE-MRI-negative gliomas. Alternatively, optimizing the physicochemical properties of FAP-targeted molecules to improve BBB penetration may enhance diagnostic accuracy and expand their applications in glioma diagnosis.

Similar to previous studies on gliomas, there was no significant correlation between tumour proliferation activity, TP53 mutations, and ^18^F-FDG PET or ^68^Ga-FAPI PET parameters 14, 30, 31. Furthermore, the difficulty in accurately delineating lesions using ^18^F-FDG PET makes it challenging to distinguish between ^18^F-FDG uptake by the tumour and normal brain. Therefore, ^18^F-FDG uptake does not accurately reflect tumour proliferative activity. GFAP is a specific glioma marker, and its expression level is positively correlated with tumour grade 32. In our study, gliomas with high GFAP expression exhibited significantly higher uptake of ^68^Ga-FAPI than GFAP-negative gliomas. We hypothesized that high-grade gliomas may coexist with high FAP and GFAP expression. However, conflicting views exist, as high GFAP expression indicates lower malignancy and higher differentiation in gliomas 33. Therefore, further validation is required. GFAP-targeted anti-tumour drugs efficiently inhibit glioma cell proliferation and promote apoptosis. Furthermore, reducing GFAP expression in the tumour tissue after treatment suggests a favourable therapeutic outcome 34. Therefore, using ^68^Ga-FAPI uptake to assess GFAP expression status may potentially guide treatment decisions and evaluate efficacy.

Our study had several limitations. First, our sample size was small, precluding significant differences in primary glioma detection rates between ^68^Ga-FAPI-46, ^18^F-FDG PET/CT, and CE-MRI. Second, our cohort's limited number of grade III gliomas hindered our ability to provide highly reliable analyses of the differences in tracer uptake between grade III and grades I-II or IV gliomas. Additionally, the uneven distribution of samples, with more than half of high-grade gliomas, resulted in a higher sensitivity for ^68^Ga-FAPI-46 PET. Increasing the number of low-grade gliomas, particularly those with low FAP expression, would likely reduce the sensitivity of ^68^Ga-FAPI-46 PET in diagnosing gliomas. Finally, our analysis based on molecular expression data was derived from a limited number of samples, constraining our capacity to draw definitive biological conclusions based on ^18^F-FDG or ^68^Ga-FAPI-46 uptake parameters.

## Conclusions

^68^Ga-FAPI-46 PET/CT outperformed ^18^F-FDG PET/CT and CE-MRI in diagnosing glioma recurrence, although the results were not statistically significant owing to the limited number of patients. For primary diagnosis, ^68^Ga-FAPI-46 PET/CT, despite the better TBR, did not surpass ^18^F-FDG PET/CT and CE-MRI in sensitivity and specificity. However, ^68^Ga-FAPI-46 PET/CT is superior to ^18^F-FDG for visualising and classifying gliomas. Therefore, its use may help improve glioma classification, evaluate recurrent tumours, and assess the nature of suspected gliomas that present as space-occupying brain lesions. Further studies from multiple centres are required to validate the potential clinical applications of ^68^Ga-FAPI-46 PET/CT imaging for glioma evaluation.

## Supplementary Material

Supplementary figures and tables.

## Figures and Tables

**Table 1:** Basic characteristics of patients.

Characteristic			No. of patients (n = 48)
Age (years)	Median (IQR)		51 (44-67)
Gender	Male/Female		32/16
Aim for PET/CT			
Space-occupying lesions			30 (63%)
	Gliomas		22 (46%)
		WHO grade I-II	7
		WHO grade III	4
		WHO grade IV	11
	Other primary CNS diseases		3 (6%)
		Meningioma	1
		Anaplastic meningioma	1
		Germ cell tumour	1
	Benign diseases		5 (10%)
		Tuberculous meningitis	2
		Brain parenchymal inflammation	1
		Cerebellar haematoma	1
		Intracranial dermoid cysts	1
Suspected recurrent gliomas			18 (37%)
		Tumour recurrence	13
		Negative for recurrence	5

**Table 2:** Diagnostic performance of PET/CT and CE-MRI for initial diagnosis of brain gliomas.

Modality	Sensitivity (95% CI)	Specificity (95% CI)	Accuracy (95% CI)
^68^Ga-FAPI-46 PET/CT	95% (21/22) [77%─100%]	40% (2/5) [5%─85%]	85% (23/27) [66%─96%]
^18^F-FDG PET/CT	77% (17/22) [55%─92%]	100% (5/5) [48%─100%]	81% (22/27) [62%─94%]
CE-MRI	100% (22/22) [85%─100%]	20% (1/5) [1%─72%]	85% (23/27) [66%─96%]
P-value (^68^Ga-FAPI-46 vs. ^18^F-FDG)	0.13	0.25	0.04
P-value (^68^Ga-FAPI-46 PET/CT vs. CE-MRI)	0.99	0.99	0.48

**Table 3:** Characteristics and differences in tracer uptake among treatment-naïve gliomas of different WHO grades.

		Grade I-II		Grade III		Grade IV			*P*-value		Overall distribution	*P*4
PET tracer	Parameters		*P*1		*P*2		*P*3	Grade I-II vs. III	Grade III vs. IV	Grade IV vs. I-II		
^18^F-FDG	SUVmax	7.74 (5.69─10.74)		11.60 (10.88─13.76)		10.33 (8.55─13.44)		0.16	0.41	0.18	10.11 (7.75─13.45)	
	TBR	1.41 (1.12─1.61)		1.45 (1.37─1.86)		1.73 (1.36─2.38)		0.53	0.85	0.15	1.52 (1.33─2.05)	
	*Background	6.55 (4.44─8.01)		7.42 (6.60─8.34)		6.30 (4.96─6.93)					6.51 (5.00─7.17)	
^68^Ga-FAPI-46	SUVmax	1.14 (0.98─3.19)	< 0.001	4.76 (3.26─6.30)	0.03	5.03 (3.34─6.20)	0.001	0.07	0.75	0.02	4.41 (1.58─5.69)	< 0.001
	TBR	4.57 (2.39─12.81)	0.002	26.28 (20.79─46.41)	0.03	19.24 (13.02─46.04)	< 0.001	0.11	0.49	0.07	17.50 (5.46─41.82)	< 0.001
	*Background	0.29 (0.15─0.49)		0.21 (0.05─0.37)		0.25 (0.11─0.59)					0.27 (0.06─0.43)	

*Background: the average SUV in the normal brain parenchyma; *P*1: Difference in PET parameters between ^18^F-FDG and ^68^Ga-FAPI-46 in grade I-II gliomas; *P*2: Difference in PET parameters between^ 18^F-FDG and ^68^Ga-FAPI-46 in grade III gliomas; *P*3: Difference in PET parameters between^ 18^F-FDG and ^68^Ga-FAPI-46 in grade IV gliomas; *P*4: Overall comparison of SUVmax and TBR between ^18^F-FDG PET/CT and ^68^Ga-FAPI-46 PET/CT across all glioma grades.

**Table 4:** Comparison of the accuracy of ^68^Ga-FAPI-46, ^18^F-FDG, and MRI in detecting recurrent gliomas

Modality	Sensitivity (95% CI)	Specificity (95% CI)	Accuracy (95% CI)
^68^Ga-FAPI-46 PET/CT	100% (13/13) [75%─100%]	100% (5/5) [48%─100%]	100% (18/18) [81%─100%]
^18^F-FDG PET/CT	85% (11/13) [55%─98%]	80% (4/5) [28%─99%]	83% (15/18) [59%─96%]
CE-MRI	100% (13/13) [75%─100%]	40% (2/5) [5%─85%]	83% (15/18) [59%─96%]
P-value (^68^Ga-FAPI-46 vs. ^18^F-FDG)	0.48	0.99	0.99
P-value (^68^Ga-FAPI-46 PET/CT vs. CE-MRI)	NA	0.25	0.25

NA: Not Applicable.

## Abbreviations

ANTs: advanced normalization tools
ATRX: alpha-thalassemia mental retardation X-linked
CE: contrast-enhanced
CNS: central nervous system
CT: computed tomography
EGFR: epidermal growth factor receptor
FAPI: fibroblast-activation protein inhibitor
FDG: ^18^F-fluorodeoxyglucose
FET: ^18^F-fluoroethyl-L-tyrosine
FAP: fibroblast-activation protein
FLAIR: fluid-attenuated inversion recovery
GE: general electric
GFAP: glial fibrillary acidic protein
IHC: immunohistochemistry
IDH: isocitrate dehydrogenase
MRI: magnetic resonance imaging
MRS: magnetic resonance spectroscopy
MPRAGE: magnetization-prepared rapid gradient echo
NF: neurofibromin
PET: positron emission tomography
ROI: region of interest
SUVmax: maximum standardized uptake value
TBR: tumour-to-background ratio
TP53: tumour protein p53
WHO: World Health Organization
WM: white matter
