# Supplementary material for: 68Ga-FAPI-46 PET/CT in the evaluation of gliomas: comparison with 18F-FDG PET/CT and contrast-enhanced MRI
Source: Theranostics. 2024 Oct 21;14(18):6935–46. doi: 10.7150/thno.103399 (PMC11610146; doi:10.7150/thno.103399)
Supplement: Supplementary file 1 — Supplementary figures and tables. [file thnov14p6935s1.pdf]

Supplementary Information

^{68}Ga-FAPI-46 PET/CT in the evaluation of gliomas: comparison with ^{18}F-FDG PET/CT and contrast-enhanced MRI

Table S1. Results of ^{68}Ga-FAPI-46 PET, ^{18}F-FDG PET, and CE-MRI in other primary CNS and benign diseases

Patient ID			^{68}Ga-FAPI-46	^{18}F-FDG	CE-MRI
	Other primary CNS diseases				
Patient 1		Germ cell tumour	Positive	Negative	Positive
Patient 2		Meningioma	Positive	Negative	Positive
Patient 3		Anaplastic meningioma	Positive	Positive	Positive
	Benign disease				
Patient 4		Cerebellar haematoma	Negative	Negative	Positive
Patient 5		Brain parenchymal inflammation	Positive	Negative	Positive
Patient 6		Tuberculous meningitis	Positive	Negative	Positive
Patient 7		Tuberculous meningitis	Positive	Negative	Positive
Patient 8		Intracranial dermoid cysts	Negative	Negative	Negative

Table S2 Correlation between Ki67 index, TP53 and parameters

		Ki67			*TP53*		
		r	*95% CI*	*P-value*	*r*	*95% CI*	*P-value*
^{68}Ga-FAPI-46	*SUVmax*	0.33	-0.22—0.72	0.24	0.19	-0.36—0.64	0.50
	TBR	-0.12	-0.60—0.42	0.67	-0.10	-0.58—0.44	0.73
^{18}F-FDG	*SUVmax*	0.03	-0.49—0.54	0.91	0.01	-0.51—0.52	0.98
	TBR	0.12	-0.41—0.60	0.66	0.03	-0.49—0.53	0.92

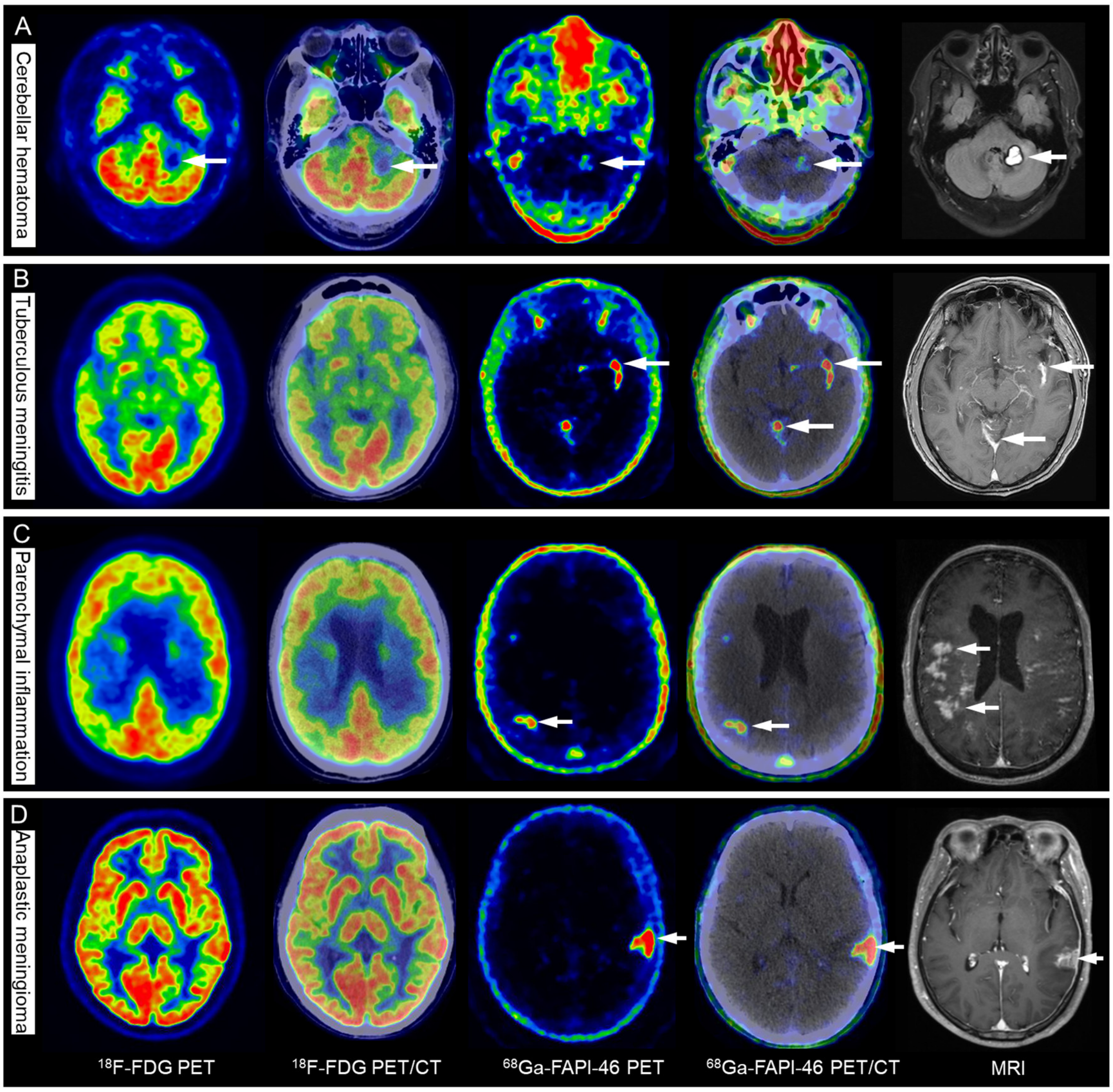

Figure S1 Findings of benign brain diseases and other primary CNS diseases on ^{68}Ga-FAPI-46 PET/CT**.** A 44-year-old male with a left cerebellar haematoma. MRI demonstrated high signal intensity in the lesion area. ^{18}F-FDG PET indicated a lack of glucose metabolism in the lesion, whereas ^{68}Ga-FAPI-46 PET demonstrated increased tracer uptake (white arrow; A). A 30-year-old male with tuberculous meningitis. MRI showed abnormal linear enhancement in the left Sylvian fissure cistern and right superior cerebellar cistern (white arrows). ^{68}Ga-FAPI-46 PET revealed multiple nodular lesions with high tracer uptake (white arrows), while ^{18}F-FDG PET showed no abnormalities (B). A 66-year-old male with brain parenchymal inflammation. MRI showed multiple enhancing lesions in the brain parenchyma (white arrows). ^{68}Ga-FAPI-46 PET demonstrated nodular high uptake foci in the right occipital lobe (white arrow), while ^{18}F-FDG PET showed no abnormalities (C). A 45-year-old male with anaplastic meningioma in the left temporal lobe. CE-MRI displayed patchy enhancement in the lesion (white arrow). PET showed high ^{68}Ga-FAPI-46 uptake (white arrow) and suspected high ^{18}F-FDG uptake in the lesion (D)

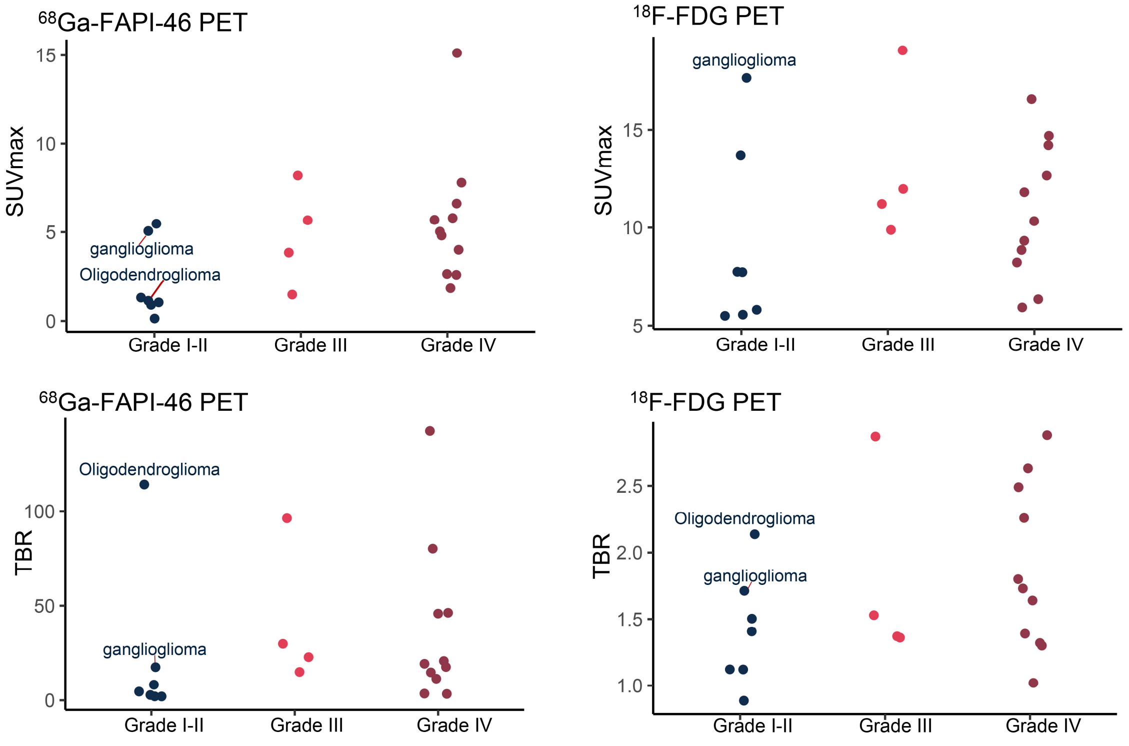

Figure S2 Uptake of [68]FAPI-46 and ^{18}F-FDG and their TBR in all newly diagnosed gliomas

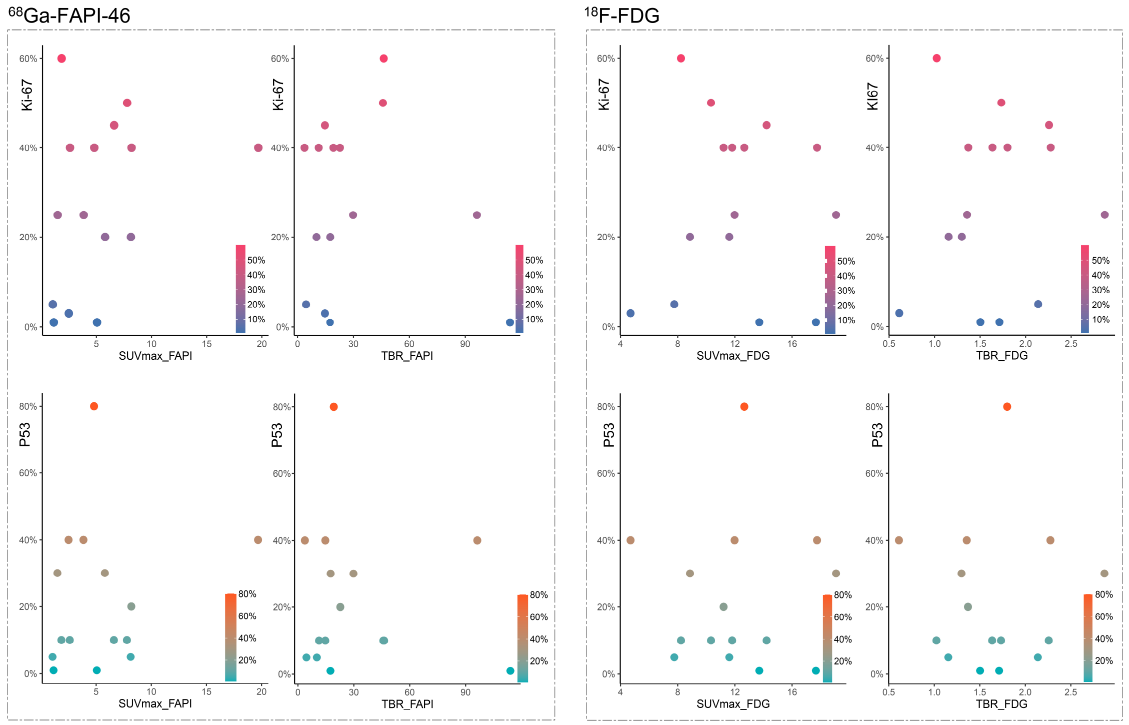

Figure S3 Scatterplot of the relationship between Ki-67 index and P53 expression score and PET parameters

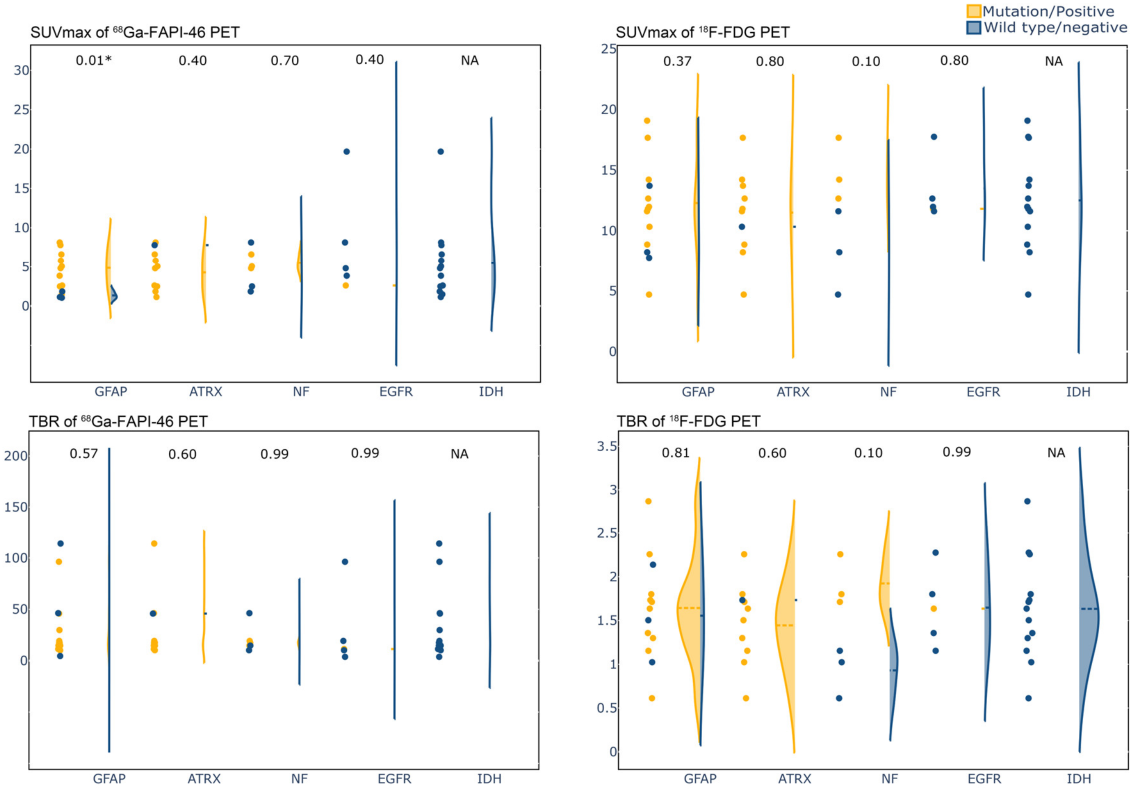

Figure S4 Relationship between biomolecular marker expression or gene changes and PET parameters in gliomas

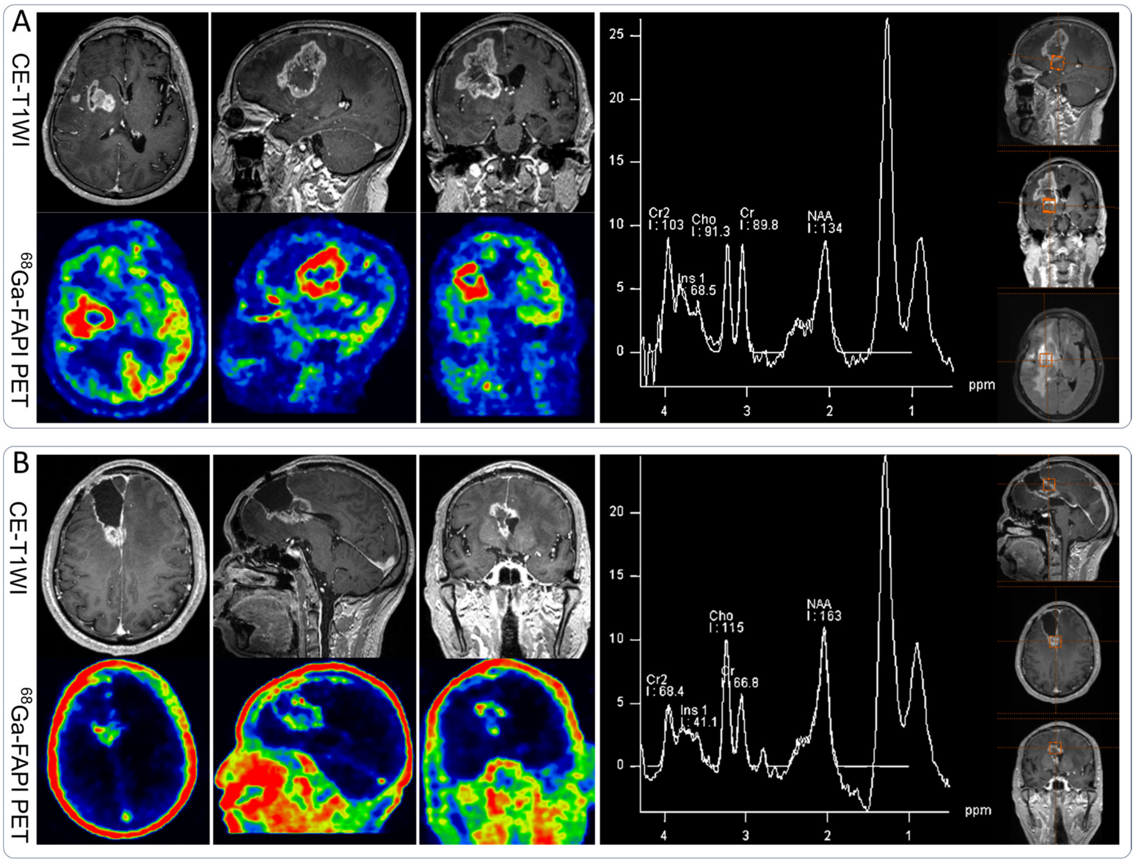

Figure S5 Abnormal enhancement was observed in the right frontal-temporal lobe and basal ganglia. MRS analysis showed a Cho/NAA ratio of 0.68, suggesting a high likelihood of radiation-induced encephalopathy based on imaging diagnosis. ^{68}Ga-FAPI-46 PET showed intense tracer uptake in the lesion. The patient passed away due to tumour recurrence (A). In another case, a ring-enhancing lesion was found below the resection cavity in the right frontal lobe and in the anterior corpus callosum. MRS analysis showed a Cho/NAA ratio of 0.7, suggesting a high likelihood of radiation-induced encephalopathy. ^{68}Ga-FAPI-46 PET showed intense tracer uptake in the lesion. Follow-up imaging indicated tumour recurrence
